# Recognizing Trained and Untrained Obstacles around a Port Transfer Crane Using an Image Segmentation Model and Coordinate Mapping between the Ground and Image

**DOI:** 10.3390/s23135982

**Published:** 2023-06-27

**Authors:** Eunseop Yu, Bohyun Ryu

**Affiliations:** Department of Plant Engineering Center, Institute for Advanced Engineering, Yongin-si 11780, Republic of Korea; yes89929@iae.re.kr

**Keywords:** port transfer crane, semantic segmentation, pseudo-LiDAR, obstacle detection

## Abstract

Container yard congestion can become a bottleneck in port logistics and result in accidents. Therefore, transfer cranes, which were previously operated manually, are being automated to increase their work efficiency. Moreover, LiDAR is used for recognizing obstacles. However, LiDAR cannot distinguish obstacle types; thus, cranes must move slowly in the risk area, regardless of the obstacle, which reduces their work efficiency. In this study, a novel method for recognizing the position and class of trained and untrained obstacles around a crane using cameras installed on the crane was proposed. First, a semantic segmentation model, which was trained on images of obstacles and the ground, recognizes the obstacles in the camera images. Then, an image filter extracts the obstacle boundaries from the segmented image. Finally, the coordinate mapping table converts the obstacle boundaries in the image coordinate system to the real-world coordinate system. Estimating the distance of a truck with our method resulted in 32 cm error at a distance of 5 m and in 125 cm error at a distance of 30 m. The error of the proposed method is large compared with that of LiDAR; however, it is acceptable because vehicles in ports move at low speeds, and the error decreases as obstacles move closer.

## 1. Introduction

Container yard congestion is a bottleneck in the flow of port logistics. Ineffective planning of loading, unloading, and storage of import and export containers as well as frequent flow of trucks can severely affect yard efficiency. To overcome this problem, transfer cranes, which were previously operated manually, are increasingly being automated. Moreover, congestion at container yards can increase the risk of accidents. Therefore, LiDAR sensors are incorporated into automated transfer cranes for recognizing nearby obstacles. 

LiDARs can be categorized as two-dimensional (2D) and three-dimensional (3D). Currently, 2D LiDARs are commonly used for detecting obstacles around cranes. However, although 2D LiDARs can detect obstacles in nearby areas, they cannot distinguish between a truck transporting a container and a stationary container. Therefore, port cranes either stop their operation unconditionally or move slowly when an obstacle is detected, regardless of the type of the obstacle; this reduces work efficiency.

Unlike 2D LiDARs, 3D LiDARs can acquire high-density 3D point cloud data. Furthermore, they can recognize the position and type of obstacles if a point cloud data-based 3D object detection deep learning model [1,2] is used. However, to detect obstacles around the gantry legs, the main collision points of the transfer cranes in the yard, we need to install a lidar on each gantry leg. As each transfer crane has four legs, this means installing a minimum of four lidars per crane. Therefore, 3D LiDARs do not have an advantage over cameras when considering obstacle recognition performance and cost.

With the maturation of camera technology and the development of autonomous vehicle-related technology, deep learning models have been incorporated for recognizing the position and type of obstacles around the vehicle based on real-time images captured by the camera. An image-based deep learning model for object detection [3,4,5] can be used to recognize the position and type of the trained object in the image. Therefore, the model can directly be applied to the camera installed in an existing crane without installing additional sensors. However, because the object in the image is recognized in the form of a bounding box, the exact outline of the obstacle cannot be recognized. Furthermore, the position of the obstacle cannot be estimated because it is recognized on the UV coordinate system of the camera, and the model cannot recognize obstacles it has not been trained on.

Unlike image-based object detection models, an image segmentation deep learning model [6,7,8] recognizes objects and classes in pixel units. Therefore, the model can recognize the outline of an obstacle. However, unlike image-based object detection models, the actual position of the obstacle cannot be recognized. To overcome problems associated with the extraction of the position of a recognized object based on an image, pseudo-LiDAR was introduced.

In stereo vision [9,10], the distance is estimated using the position error of feature points extracted from each image obtained by two cameras that have exact information regarding each other’s position. Applying stereo vision to transfer cranes requires installation of additional equipment. However, transfer cranes need to operate 24 h a day. Therefore, stopping the operation of a transfer crane to install equipment is not feasible.

In a time-of-flight (ToF) camera [11], the distance is measured by emitting infrared rays and subsequently calculating the time the infrared rays take to reach the obstacle and return to the image sensor after reflection from the obstacle. However, similar to stereo vision, ToF cameras require installation of additional equipment. Furthermore, the effective distance is short because of the characteristics of infrared wavelengths. Furthermore, outdoor noise due to sunlight renders the application of the ToF camera to transfer cranes difficult. 

A depth estimation model [12,13,14,15] was trained on camera images and high-density LiDAR point cloud data to predict the depth of each pixel of an image when the image was inputted. For the depth estimation model, existing cameras can be used without installing additional equipment. However, generating training data for the depth estimation model requires the installation of a high-precision LiDAR on the crane to collect long-term data. To solve the problem of inadequate data, publicly available training data can be used. However, most of the models and training data [16] that are publicly available cater to autonomous vehicles. Compared with that of port transfer cranes, the surrounding and installation environments of autonomous vehicles differ considerably. Therefore, using publicly available training data for port transfer cranes is inadequate.

In this study, a novel method was proposed for estimating the position and type of trained and untrained obstacles around the crane by applying a table that transforms the image coordinates to ground coordinates and a semantic segmentation model trained on the ground images obtained using a camera installed in an existing transfer crane. The proposed method has the following advantages: Compared with the conventional camera-based obstacle recognition technique, the proposed method can recognize the position of an obstacle using only a camera by applying the coordinate conversion table. Moreover, the method can recognize both trained and untrained obstacles by training on ground images in addition to the main obstacles. Compared with the conventional pseudo-LiDAR-based obstacle recognition technique, the installation of additional equipment is not required because in the proposed method, a camera already installed on the crane is used. Furthermore, obtaining data is easy because the proposed method only requires camera images as the training data.

The remainder of this paper is organized as follows: Section 2 explains the process and general functions of the proposed method. Section 3 describes the data collection and preprocessing method. Section 4 explains how the semantic segmentation model trained on the ground recognizes trained and untrained obstacles. Section 5 discusses the method of creating a table that converts the image coordinates to the ground coordinates and the results of applying this method. The estimation of the position of obstacles using the proposed method and the position of obstacles measured with a LiDAR are compared in Section 6. Finally, Section 7 presents the conclusions of this study and discusses future research directions.

## 2. System for Detecting Obstacles around the Transfer Crane

**Figure 1:**
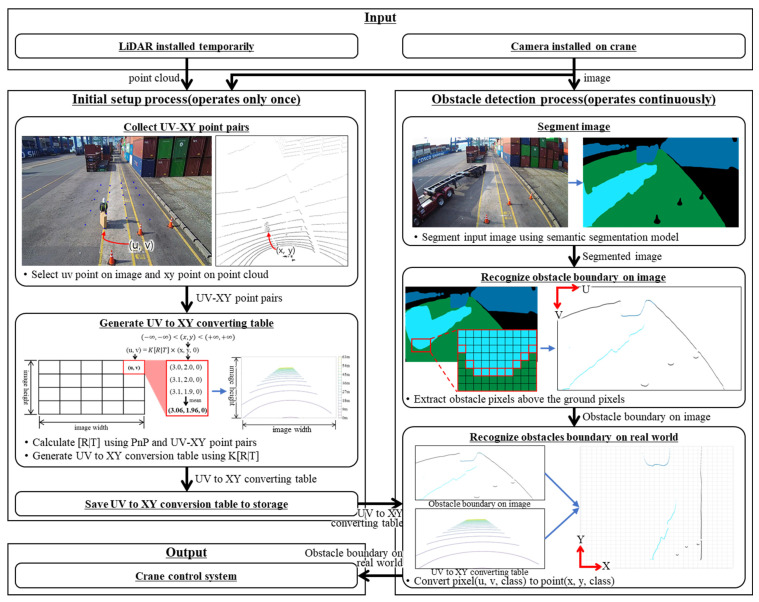
Obstacle detection process around the transfer crane.

Figure 1 displays the process of the system for detecting obstacles around the transfer crane. This process consists of the initial setup process, which is executed only when the obstacle detection system is first installed on the crane, and the obstacle detection process, which runs when the crane is operated.

The initial setup process creates a table that converts the UV coordinates of the image to the XY coordinates of the ground. First, LiDAR is temporarily installed to collect real coordinates. The UV coordinates from the image captured with the camera installed on the crane are collected, and the XYZ coordinates corresponding to the UV coordinates are collected from the LiDAR point cloud. These coordinates are then used to create a list of UV–XYZ coordinate pairs. Next, the UV–XYZ coordinate pair list and the perspective-n-points (PnP) [17] algorithm are used to calculate the rotation transformation matrix and translation transformation matrix, which indicate the positional relationship between the UV coordinate system of the camera image and the XYZ coordinate system of the LiDAR point cloud. Finally, a table is created that converts the UV coordinates of the camera image to the XY coordinates of the ground using transformation matrices.

In the obstacle detection process, an obstacle is recognized in the camera image and the type and position of the obstacle are estimated on the XY coordinate system of the ground by applying the coordinate conversion table created in the initial setup process. First, the semantic segmentation model recognizes the class of the image pixels. To recognize the image coordinate system-based position of the obstacle from the recognition result, the boundary of the obstacle is recognized by applying a filter that extracts pixels in which the class of the candidate pixel (*u*,*v*) being checked is not the ground and the class of the pixel below it (*u*,*v* + 1) is the ground. Finally, the position and type of trained obstacles and the position of untrained obstacles are recognized on the XY coordinate system of the ground by applying the coordinate conversion table created in the initial setup process to the obstacle boundary information on the UV coordinate system of the image.

## 3. Data Collection

**Figure 2:**
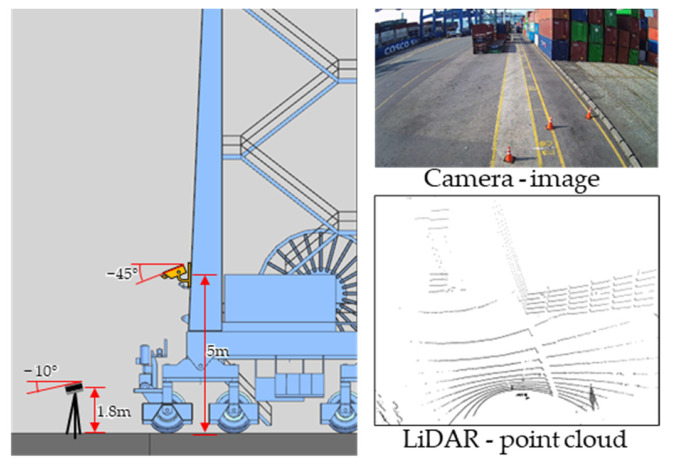
Installation of the camera and LiDAR for data collection.

A camera and LiDAR were used to collect data for generating the training dataset and verifying the proposed method. For the camera, we used a camera that was already installed on the crane. LiDAR was installed using a tripod on the ground in front of the crane. For easy detection of vehicles approaching from a distance and to make a correction by recognizing the ground so that the ground and the XY-plane of the LiDAR coordinate system are parallel, LiDAR was installed facing the ground at approximately −10°. The specifications and installation information of the camera and LiDAR used in the experiment are presented in Table 1 and Figure 2.

When an obstacle entered the detection area after installation of the camera and LiDAR, data were collected by saving the image data and point cloud data at the operation cycle of each sensor and recording the time the data were saved. During the data acquisition, 48 trucks and 6 people passed through the detection area. Then, we collected image data with a time interval of at least 3 s between images to avoid training the model with the same images.

To verify a mapping table for converting the UV coordinates of the camera image to the XY coordinates of the ground using the collected data, the following preprocessing was performed:

To create a mapping table, the XY-plane of the point cloud data coordinate system should coincide with the ground. However, the coordinate system of the point cloud data collected using the LiDAR sensor conformed to the coordinate system of the LiDAR sensor. Therefore, calculating the rotation and translation transformation matrices that make the XY-plane of the point cloud data coincide with the ground was necessary. 

First, only the points that corresponded to the ground were extracted from the point cloud data. The normal vector of the ground was then calculated assuming that the extracted points were on a plane. Next, the point cloud data were rotated so that the normal vector of the ground and the Z-axis of the LiDAR coordinate system were parallel. Then, the point cloud data were translated in the Z-axis direction by a distance that corresponded to the shortest distance between the origin of the point cloud data and the plane corresponding to the ground so that the XY-plane of the point cloud data coordinate system coincided with the ground. Finally, after calculating the positional relationship between the camera and LiDAR using the PnP algorithm, the point cloud data—whose origin is the position where the LiDAR was installed $\left(x_{L},\;y_{L},\;0\right)$—was transformed by translating it to the position $\left(x_{C},\;y_{C},\;0\right)$ where the camera was installed.

To verify the proposed method in this study, the distance estimated by the camera and LiDAR sensor should be compared. However, because the operation cycle and input delay time of the two sensors differed considerably, comparing the data collected from the camera and LiDAR and matching the data obtained at the same or similar time was necessary. First, the point cloud data were overlapped on the image. The corners of the moving truck were then visually observed to evaluate whether the image and point cloud data matched. Then, 500 ms was added to saved time data of the LiDAR to synchronize time of camera and LiDAR.

## 4. Image Semantic Segmentation and Obstacle Boundary Extraction

In this study, a semantic segmentation model was used to recognize the type and outline of obstacles in camera images. The object detection model—a deep learning model widely used to recognize objects in images—recognizes the type and estimates the position of an object in the image in the form of a bounding box. However, the model cannot recognize the exact outline of the object. Segmentation models are often used for recognizing complex object shapes because they can discern the exact contours of objects by recognizing them at the pixel level [18,19,20]. In addition to recognizing objects, the semantic segmentation can also identify their classes [21,22].

Generally, classes are assigned to almost all pixels of the training data for the semantic segmentation model. Unlike the training data of the object detection model, which represents the position of an obstacle with a bounding box, the outline of an obstacle should be depicted in detail in pixels. Various types of objects, such as containers, ships, trucks, cranes, people, and cars, exist in a port. However, labeling all objects existing in a port with class labels is time consuming. Therefore, to reduce the labeling time, the labeling classes were limited as follows. People, cars, trucks, and containers, obstacles often included in the training data, were labeled as separate classes. However, obstacles that are often included in the training data but are not of interest or obstacles that were infrequently included, such as sky, ships, unpaved ground, and cranes on another line, were labeled as misc. class. Furthermore, for the recognition of untrained obstacles on the ground, we labeled the paved ground where cranes and major obstacles move around, even though the paved ground is not an obstacle.

**Figure 3:**
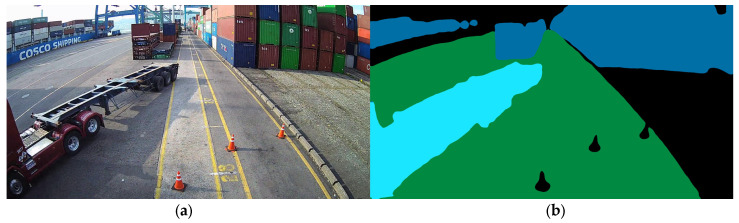
Input image (**a**) and result of semantic segmentation (**b**).

Considering the limited training data, we selected HRNetV2 + OCR [23,24], which is focused on recognition accuracy rather than image processing speed, as the deep learning model for recognizing obstacles in images. A comparison of the performance of HRNetV2 + OCR and the real-time targeted model on the Cityscapes dataset is shown in Table 2. Out of 200 labeled images, 160, 30, and 10 were randomly selected for training, validation, and testing, respectively. Figure 3 displays the results of recognizing the class for each pixel using the trained model.

**Figure 4:**
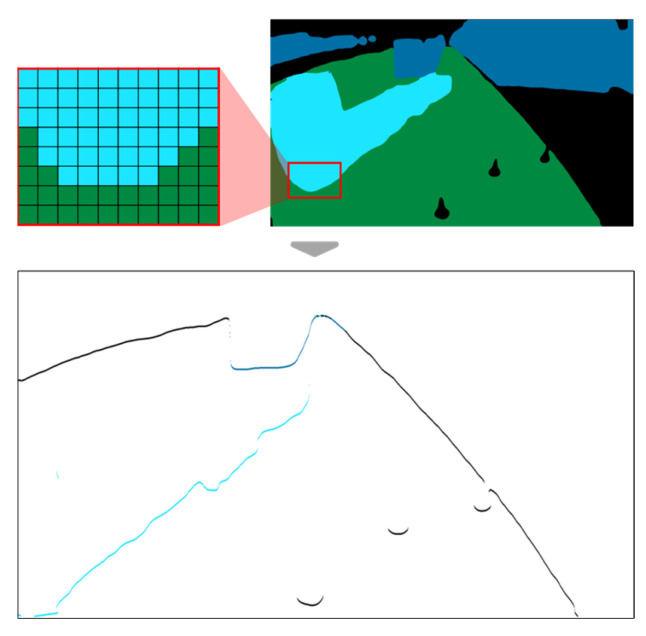
Extraction of obstacle boundary from the segmented image.

Given that all obstacles in the port are on the ground, the method of extracting the boundary of an obstacle from the semantic segmentation result by applying pixel filtering was as follows: The filter recognizes the pixel (*u*,*v*) being checked as the boundary of an obstacle if the pixel does not belong to the ground class, and the pixel below it (*u*, *v* + 1) belongs to the ground class. Figure 4 displays the result of extracting the boundary by applying the filter to the image in Figure 3b.

## 5. Generation of the Coordinate Conversion Table

Since the boundaries of the recognized obstacles (Section 4) were expressed in the UV coordinate system, the UV coordinates had to be converted to XY coordinates to recognize the location of the obstacles. In this study, we used the PnP algorithm to calculate the position relationship between the camera and the LiDAR and used position relationship to create a conversion table from UV coordinates to XY coordinates.

To calculate the position relationship of the camera and LiDAR using the PnP algorithm, at least three UV–XYZ coordinate pairs are required. First, we placed a rectangular paper box with the corners facing the camera and LiDAR at random points and obtained the image and point cloud pair dataset. Then, we extracted the corresponding UV and XYZ coordinates from the dataset.

The UV coordinates were extracted by the user clicking on the corner of the box in the image where it touched the ground. The XYZ coordinates were extracted by automatically selecting the point with the smallest Y-value in the point cloud corresponding to the box, because it is difficult for the user to directly select it from the point cloud. The following method was used to automatically extract XYZ coordinates from the point cloud data.

**Figure 5:**
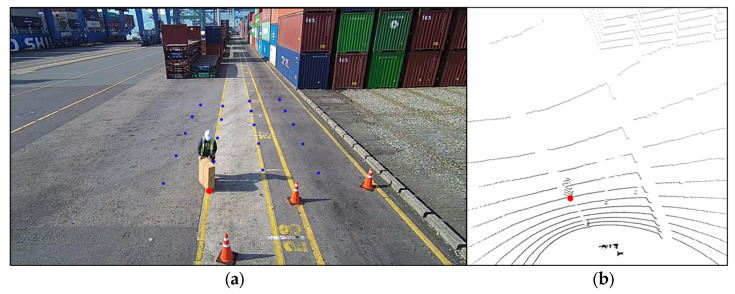
Camera image (**a**) and LiDAR point cloud data (**b**) for acquiring UV–XY0 coordinate pairs.

To calibrate the coordinate system so that the XY-plane of the LiDAR is parallel to the ground, we extracted only the points corresponding to the ground and calculated the plane equations. Then, we calculated a matrix to transform the plane to match the XY-plane of the LiDAR. The transformation matrix was applied to all points of the LiDAR point cloud data. After the calibration of the LiDAR coordinate system, points with a Z-value of 10 cm or less were removed from the point cloud data to filter only the points that corresponded to the box. The point with the smallest Y-value was automatically filtered from the remaining points, and the XY0 coordinates were selected, as indicated by the red circle in Figure 5b. The tasks were repeated to obtain 20 pairs of UV–XY0 coordinates located between 6 and 17 m in front of the crane. The obtained coordinate pairs are represented by the blue and red dots in Figure 5.

Knowing the camera matrix *K* and the position relationship matrix *R*|*T* between the camera and LiDAR, as shown in Equation 1, a point (*x*,*y*,*z*) in the LiDAR point cloud coordinate system can be converted to a point (*u*,*v*) in the camera image coordinate system. Camera matrix *K* can be calculated easily through chessboard pattern recognition. The transformation matrix *R*|*T* was calculated using the UV–XY0 coordinate pairs obtained from the port and the PnP calculation function provided by OpenCV. Equation 1 can be used to convert (*x*,*y*,*z*) to (*u*,*v*), but cannot convert (*u*,*v*) to (*x*,*y*,*z*). To solve this problem, a mapping table that converts (*u*,*v*) to (*x*,*y*,0) was generated in this study.

(1) $$\left[\begin{array}{c} u\\ v\\ 1 \end{array}\right]=K\left[R|T\right]\left[\begin{array}{c} x\\ y\\ z\\ 1 \end{array}\right]=\left[\begin{array}{ccc} f_{x} & \gamma & c_{x}\\ 0 & f_{y} & c_{y}\\ 0 & 0 & 1 \end{array}\right]\left[\begin{array}{cccc} r_{11} & r_{12} & r_{13} & t_{1}\\ r_{21} & r_{22} & r_{23} & t_{2}\\ r_{31} & r_{32} & r_{33} & t_{3} \end{array}\right]\left[\begin{array}{c} x\\ y\\ z\\ 1 \end{array}\right]$$

**Figure 6:**
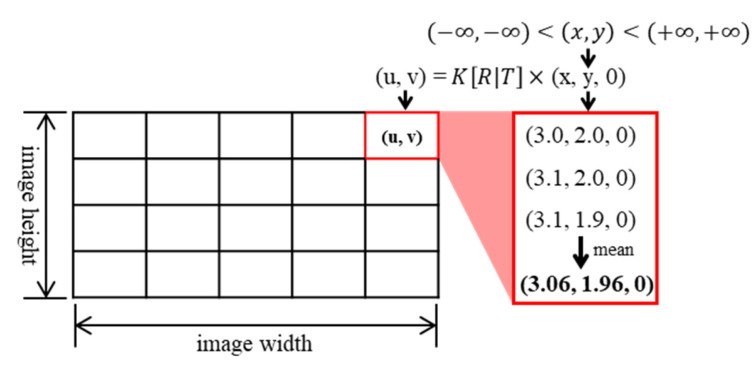
Generate UV–XY0 conversion table.

Figure 6 illustrates the method of generating a mapping table for UV–XY0 coordinate conversion.

Step 1: Create a matrix equal to the size of the image.

Step 2: Enter (*x*,*y*,0) coordinates of a sufficiently wide range in the matrix calculated using PnP to calculate (*u*,*v*) coordinates that correspond to the (*x*,*y*,0) coordinates entered. Next, store the (*x*,*y*,0) coordinates in the (*u*,*v*) components of the matrix created above.

Step 3: Calculate the average of several (*x*,*y*,0) coordinates stored in each component of the matrix and store the average value in each component.

**Figure 7:**
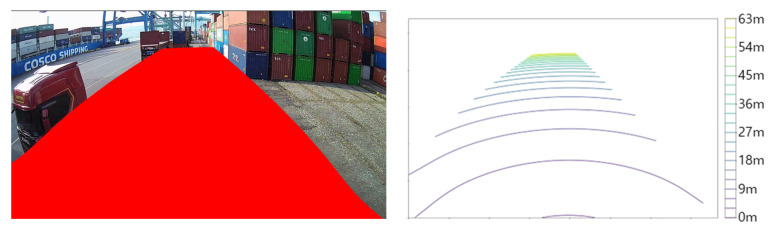
Coordinate conversion table area (**left**) and distance visualization (**right**).

A width of 16 m and a length of 60 m were set as the range in this study, considering the direction of the crane movement and the movement of objects likely to be detected to prevent a collision. Figure 7 displays the effective range of the mapping table.

**Figure 8:**
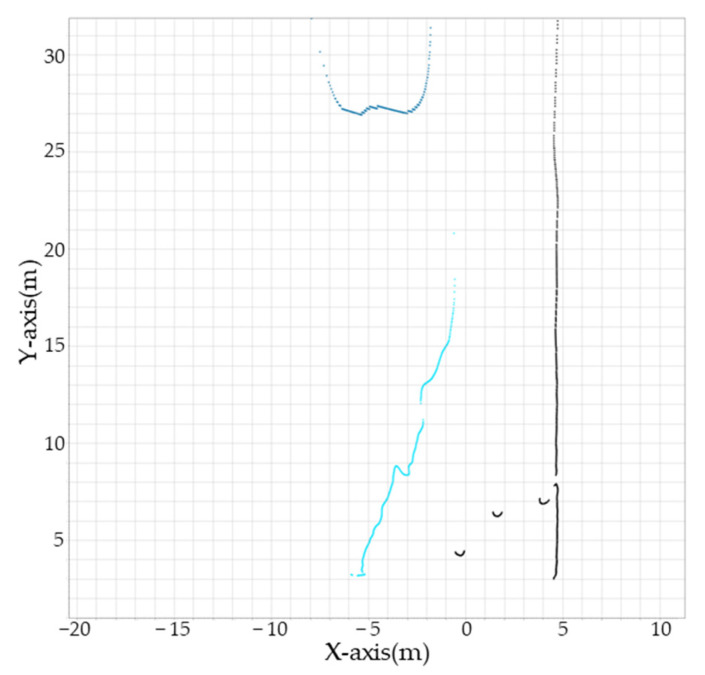
Result of the conversion to 2D point cloud data including class information.

Finally, by applying the coordinate transformation table to the image UV coordinate-system-based obstacle boundary extracted from the semantic segmentation result, the ground XY coordinate-system-based obstacle boundary can be obtained as shown in Figure 8.

## 6. Experiment

**Figure 9:**
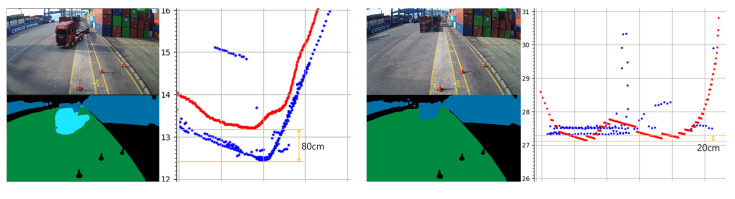

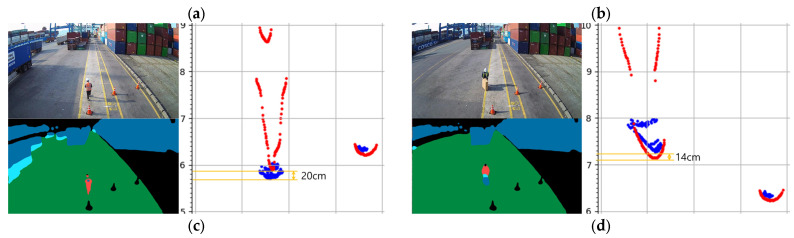
Comparison of the distance estimated by the camera and measured by the LiDAR. In the 2D point cloud images, red points are estimated using the camera and blue points are measured using the LiDAR. The obstacles are as follows: (**a**) truck (trained); (**b**) container (trained); (**c**) human (trained); (**d**) box (untrained).

As displayed in Figure 9, the distances to a truck, person, and container estimated using the camera were compared with the distances measured by the LiDAR to verify the validity of the proposed method. Considering that the collision direction of the crane and the obstacle was in the Y-axis direction, the point with the shortest distance to the Y-axis from the obstacle boundary obtained using the camera was compared with the shortest distance to the Y-axis from the obstacle boundary obtained using LiDAR.

To validate the performance of the proposed method, we collected data as 10 trucks and 3 people passed by, then compared the error between the distances estimated by our method and those measured by LiDAR when the distance to the obstacles was approximately 5, 10, 15, 20, 25, and 30 m. For trucks, the error was 32 cm at 5 m and 125 cm at 30 m. For people, the error was 22 cm at 5 m and 30 cm at 15 m, and they were not recognized in the image by the semantic segmentation phase at distances greater than 20 m due to their small size. The distance-based estimation error for trucks and people is shown in Table 3. As for the container, it did not move during data collection, and so the verification was carried out only for one container positioned at a distance of 27.3 m, as shown in Figure 9b. The estimation error for the container was 20 cm.

The distance estimated using the camera and the distance measured by the LiDAR were compared for a box that was not included in the training data to verify the recognition performance for untrained obstacles. The box was recognized as a container and a truck in the semantic segmentation phase, but the boundary of the box could be estimated using the ground recognition result. Therefore, the proposed method cannot recognize the class of untrained obstacles but can recognize their positions.

**Figure 10:**
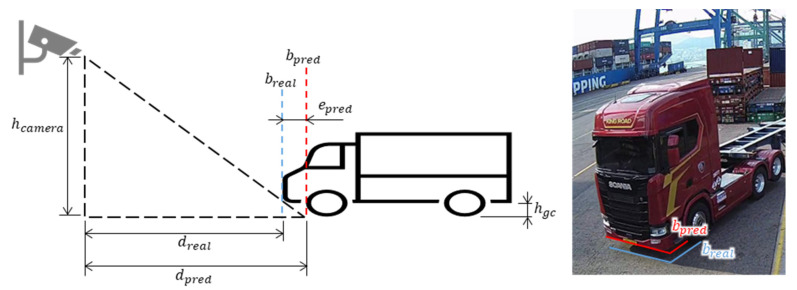
Obstacle boundary estimation error according to the camera installation height and minimum ground clearance.

The estimation error for the truck was larger than the estimation error for other obstacles (container and person) because although the truck has wheels that are attached to the ground, a space exists between the ground and the body of the truck when looking at the truck from the front or side. As displayed in Figure 10, when the truck approaches the camera head-on, the pseudo-LiDAR recognizes b_pred, the boundary between the ground and the truck from the camera’s viewpoint, as the boundary of the truck, although the actual boundary of the truck is b_real.

Theoretically, the prediction error of the proposed method reduces as the distance between the obstacle and the camera decreases. Because cranes and container trucks move at a low speed of 20 km/h or less in the port, the prediction error of the proposed method is determined to be within the allowable error range for preventing collisions.

## 7. Conclusions

In this study, a novel method was proposed for recognizing the type and position of obstacles on the ground coordinate system by applying semantic segmentation to the images captured by a camera installed on the port transfer crane. The segmentation model recognizes the class of each pixel in the image using an image filter to extract the boundary of the obstacle on the UV coordinate system of the image. A coordinate conversion table was then applied to convert the position information of the obstacles on the image coordinate system to the position information on the ground coordinate system.

To verify the method proposed in this study, the positions of the obstacles estimated using the camera were compared with the positions of the obstacles measured by the LiDAR. In the case of a truck, which is a major obstacle, the position recognition error was larger than that of other obstacles because of the minimum ground clearance. However, the error due to the minimum ground clearance decreased as the distance was reduced. Because obstacles in the port move slowly, the position recognition error of the proposed method was in the allowable error range.

This study confirmed that it is possible to recognize the classes and locations of both trained and untrained obstacles around the crane using only the cameras installed on the crane. However, we also identified several limitations in the data used and the method proposed in this study.

With regards to the data used in this study, there are several limitations. The data were acquired during a single day, and therefore, influences from environmental factors such as lighting and weather have not been considered. Moreover, the diversity of obstacle types was not extensive, leading to potential dependence on the shape and color of obstacles. Particularly for people, all individuals included in our data were wearing safety helmets and vests, which necessitates further testing to confirm whether people in different outfits can be detected accurately as well. Containers have standardized shapes, and trucks have minor differences in shape and color depending on the manufacturer, so we believe that with careful selection of training data, these limitations can be managed.

In terms of the method proposed in this study, there are also some limitations. While the proposed method can recognize the location of untrained obstacles, it cannot recognize their class. For instance, if a person is in the collision path, the crane should slow down and stop. However, if a person carrying a box is recognized as a container, as in Figure 9d, the crane would still eventually stop, but the person might be in danger. Additionally, our method assumes that obstacles exist on the ground for detection. If an obstacle is not on the ground, the estimation error for that obstacle could be very large, rendering the detection meaningless.

In the future, we plan to replace semantic segmentation used in the obstacle recognition process with panoptic segmentation to extract object information as well as class information from images. In addition, to overcome the limitations of the method proposed in this study, we are considering the use of products that include short-range LiDAR and cameras, such as the Intel RealSense L515. This not only has the disadvantage of requiring additional sensor installation, but also allows for the automation of the cumbersome initial setup process. With the use of L515, we expect it to be possible to achieve more precise location estimation for short-range obstacles, recognize obstacles that are not on the ground, and recognize people along with untrained obstacles as the person class.

## Figures and Tables

**Table 1:** Specifications of the camera and LiDAR.

	Camera	LiDAR
Product	HikVision DS-2CD2725	Velodyne Puck 16ch
Resolution	1920 × 1080	1800 × 16 (600 rpm)
FOV	105° × 56°	360° × 30°
Frequency	30 fps	10 fps

**Table 2:** Compare the performance of semantic segmentation models.

Model	mIoU	FPS
HRNetV2 + OCR [24]	84.2	-
PIDNet-L [25]	80.9	31.1
DDRNet-23 [26]	79.4	51.4
SFNet(DF2) [27]	77.8	87.6

**Table 3:** Error in obstacle distance estimation according to the distance from the obstacle.

Class	Measured Distance (m, ±0.3)	Estimation Error (m, RMSE)
Truck	5	0.32
	10	0.78
	15	0.86
	20	0.94
	25	1.04
	30	1.25
Person	5	0.22
	10	0.23
	15	0.3

## Data Availability

Not applicable.
